# Dorsal-zone-specific reduction of sensory neuron density in the olfactory epithelium following long-term exercise or caloric restriction

**DOI:** 10.1038/s41598-018-35607-w

**Published:** 2018-11-23

**Authors:** Ayinuer Tuerdi, Shu Kikuta, Makoto Kinoshita, Teru Kamogashira, Kenji Kondo, Shinichi Iwasaki, Tatsuya Yamasoba

**Affiliations:** Department of Otolaryngology, Graduate School of Medicine, University of Tokyo, 7-3-1 Hongo, Bunkyo-ku, Tokyo, 113-0033 Japan

## Abstract

Exercise (Ex) and caloric restriction (CR) reduce oxidative stress and improve organ function. For instance, voluntary Ex or CR is known to reduce age-related cochlear damage in male C57BL/6J mice. However, the effect of Ex and CR on the olfactory system is unknown. In this study, we confirmed the positive effect of Ex and CR on age-related cochlear damage, but found that Ex and CR affected negatively cell dynamics in the olfactory epithelium (OE) by reducing the number of mature olfactory sensory neurons (OSNs) and increasing the number of proliferative basal cells and apoptotic OSNs in the dorsal zone of the olfactory epithelium (OE), which contains neurons expressing NADPH quinone oxido-reductase 1 (NQO1). In addition, these interventions resulted in lower odor-induced c-fos expression in areas of the olfactory bulb receiving projections from dorsal-zone OSNs than in areas receiving ventral-zone projections. Further, we observed substantial oxidative stress in NQO1-positive cells and apoptotic OSNs in the dorsal zone in Ex and CR animals. These results suggest that, in contrast to their positive effects in other organs, Ex and CR facilitate oxidative stress and negatively impact structure and function in dorsal-zone OSNs, probably in association with NQO1 bioactivation.

## Introduction

Oxidative stress results from an imbalance between the production of reactive oxygen species (ROS) and the body’s antioxidant defenses. ROS is a general term describing chemically reactive oxygen molecules and free radicals generated through aerobic respiration as oxidative byproducts of normal cellular metabolism^1^. High levels of ROS can be lethal and can cause extensive damage to proteins, DNA, and lipids. In addition, ROS are involved in many age-related pathologies, including hearing loss, cataracts, insulin resistance, and skeletal muscle loss^1,2^. Preventing the deleterious effects of ROS can reduce oxidative damage and maintain normal cellular function and tissue homeostasis.

Long-term exercise (Ex) and caloric restriction (CR) are considered robust non-genetic methods for reducing oxidative stress in many organs^3,4^. These interventions can reduce body weight, systemic insulin/insulin-like growth factor (IGF) signaling, the basal rate of mitochondrial hydrogen peroxide production in tissue, and inflammation. The mechanisms of action of Ex and CR are similar, include modifications in energy metabolism, and provide sustained benefits to age-related health^3,5–7^. Despite the wealth of current literature, it is unclear whether these interventions uniformly benefit all organs.

The olfactory epithelium (OE) is exposed continuously to various potentially harmful air pollutants due to its anatomical location and function. In addition, olfactory sensory neurons (OSNs) are highly vulnerable to damage from endogenous neurotoxic compounds derived from normal cellular respiration and various metabolic reactions, which can induce a loss of OSNs in the OE. Loss of OSNs triggers the regeneration of new OSNs through the proliferation and differentiation of progenitor cells. These new OSNs are incorporated into olfactory circuits. However, nutritional status can affect dynamically and profoundly the cell dynamics of OSNs through cell death and the regeneration process, as well as through changes in energy metabolism and the production of metabolic byproducts at the cellular level^8,9^. These observations suggest that Ex and CR interventions affect the homeostasis of cell dynamics in the OE.

Auditory function in mice is normal in early life and declines gradually in later life^2^. Declines in auditory function are associated with loss of hair cells (HCs), but Ex and CR delayed the age-related degeneration of HCs^2^.

In the present study, we sought to confirm whether 10 months of voluntary Ex or a 20% caloric reduction can delay age-related degeneration of HCs and have a positive effect on the auditory system in male C57BL/6J mice, as reported previously^2,10^. In addition, we investigated whether 10 months of voluntary Ex or 20% caloric reduction affects the cell dynamics of OSNs in the olfactory system. Our results show that long-term Ex or CR prevents age-related degeneration of the cochlea, and has a positive effect on the auditory system. However, Ex or CR had negative structural and functional effects in the dorsomedial area of the OE, as determined by the co-localization of these effects with the endocellular enzyme, NADPH quinone oxido-reductase 1 (NQO1). Furthermore, oxidative stress was observed in NQO1-positive cells and apoptotic OSNs, probably in association with the bioactivation of NQO1.

## Results

**Figure 1:**
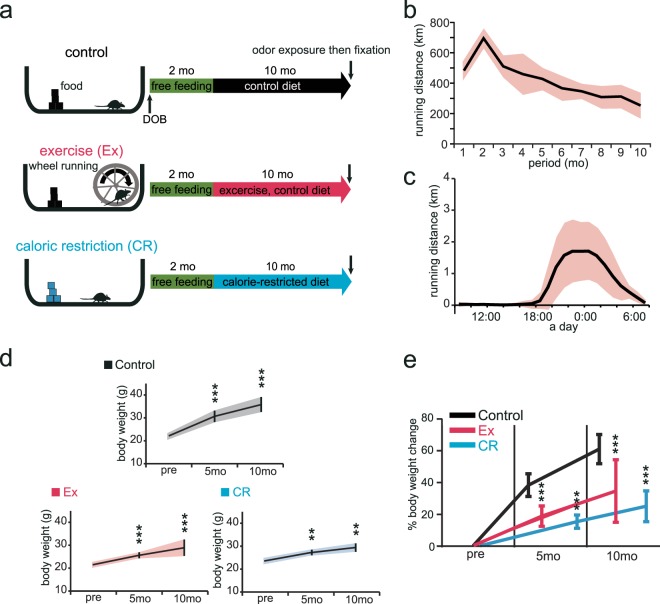
Effects of exercise or calorie restriction on body weight. **(a)** Study design. At 2 months of age, mice were divided into three groups: Ex, voluntary exercise on a running wheel with the control diet for 10 mo; CR, calorie-restricted diet for 10 mo. All mice were exposed to odors prior to fixation for subsequent measurement of c-fos expression. DOB, date of birth. **(b)** Time course of wheel-running distance (km/mo) for the 10 mo experimental period (n = 8 mice, mean ± SD). **(c)** Daily mean wheel-running distance. Wheel-running exercise followed a regular daily cycle. **(d)** Body weight at 2 mo (pre), 5 mo, and 10 mo in each group (n = 8 mice per group). Body weight in each group significantly increased after 5mo compared with 2mo (pre), and further significantly increased after 10 mo (**p < 0.01; ***p < 0.001; Steel test). **(e)** Percentage change in body weight from 2 mo (pre) to 5 mo and 10 mo (n = 8 mice per group). There was a significant reduction in percentage body-weight change at 5 mo and 10 mo in Ex and CR mice compared with control mice (*p < 0.05; **p < 0.01; ***p < 0.001; Mann-Whitney U test).

Figure 1 illustrates the experimental design for Ex and CR. Mice were allocated randomly into the following three groups at 2 months of age: control, Ex, and CR groups (Fig. 1a). Running activity in Ex mice peaked at 2 months and, then, decreased gradually over time (Fig. 1b). Ex was performed daily (Fig. 1c). The body weight of control, Ex, and CR mice (n = 8 mice per group) increased significantly over the 10 month experimental period [(Control: 5 months, p < 0.001; 10 months, p < 0.001) (Ex: 5 months, p < 0.001; 10 months, p < 0.001) (CR: 5 months, p < 0.01; 10 months, p < 0.01) (Steel test; Fig. 1d)]. However, the percentage change in body weight was significantly different between the groups. Body-weight increase in mice in the experimental groups was significantly lower than that in their age-matched controls [(5 months: Ex, p < 0.001; CR, p < 0.001) (10 months: Ex, p < 0.001; CR, p < 0.001) (Mann-Whitney U test; Fig. 1e)]. These results suggest that Ex and CR reduce body-weight gain, which can influence metabolic energy systems.

### Ex and CR delay the loss of outer HCs associated with age-related cochlear degeneration

**Figure 2:**
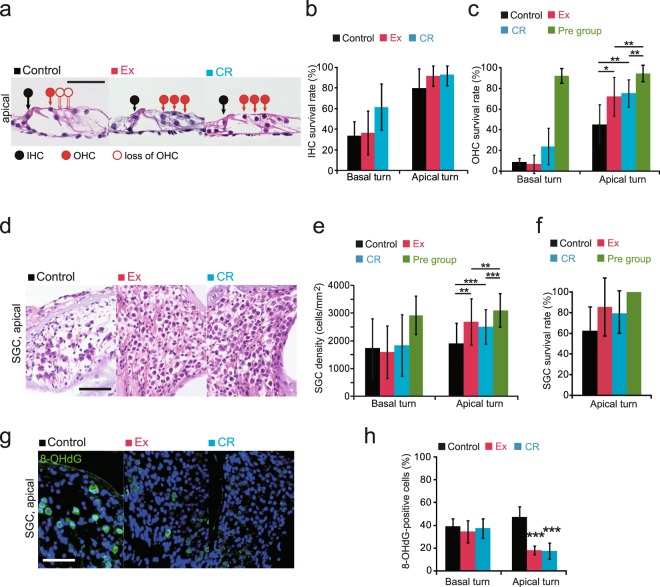
Effects of 10 month Ex, PR, and CR on age-related degenerative changes of inner ear. **(a)** Hematoxylin and eosin-stained images at the apical turn of the organ of Corti in control, Ex and CR mice. Black circles, IHCs; red circles, OHCs; open circles, loss of HCs; and closed circles, intact HCs. Scale bar, 50 μm. **(b)** IHC survival rates in control, Ex and CR mice. There were no significant differences in IHC survival rate in Ex or CR mice compared with control mice at either the basal turn or the apical turn (Mann-Whitney U test). **(c)** OHC survival rates in control, Ex, CR and Pre mice. There was a significant increase in OHC survival rate in Ex and CR mice at the apical turn compared with control mice (*p < 0.05; **p < 0.01; Mann-Whitney U test). The OHC survival rates of Ex and CR mice did not return to those of pre mice (**p < 0.01; Mann-Whitney U test). **(d)** Representative images of spiral ganglion cells (SGCs) at the apical turn in control, Ex and CR mice. Scale, 50 μm. **(e)** Summary of SGC density at the basal and apical turns. Mean SGC densities in Ex and CR mice at the apical turn were higher than those in control mice (**p < 0.01; ***p < 0.001; Mann-Whitney U test). The SGC densities in Ex and CR mice did not return to those of pre mice (**p < 0.01; ***p < 0.001; Mann-Whitney U test). **(f)** SGC survival rates in control, Ex, CR and Pre mice. **(g)** Representative images of 8-OHdG-positive cells at the apical turn in control, Ex and CR mice. Scale, 50 μm. **(h)** Summary of 8-OHdG-positive cells at the basal and apical turns. There was a significant decrease in mean 8-OHdG-positive cells in Ex and CR mice at the apical turn compared with control mice (***p < 0.001; Mann-Whitney U test).

We examined whether the experimental regimes could induce positive effects within the auditory system, as reported previously^11^. Age-related decline in auditory function is associated with the loss of inner HCs (IHCs), outer HCs (OHCs), and spiral ganglion cells (SGCs) in the basal turn, and the progressive loss of OHCs and SGCs in the apical portion of cochlea^2^. We observed histological changes in IHCs, OHCs, and SGCs in control, Ex, and CR mice (Fig. 2). We calculated the survival rates of IHCs and OHCs to determine the number of surviving HCs in the organ of Corti (see Materials and Methods for more details). We observed a greater extent of age-related degeneration of HCs in the basal turn than in the apical turn (Fig. 2a and c). Age-related degeneration in OHCs, but not in IHCs, was prevented in the apical turn of Ex and CR mice [(IHCs in basal turn: Ex, p = 0.44; CR, p = 0.26) (IHCs in apical turn: Ex, p = 0.11; CR, p = 0.17) (OHCs in basal turn: Ex, p = 0.67; CR, p = 0.34) (OHCs in apical turn: Ex, p < 0.05; CR, p < 0.01) (n = 4 mice per group; Mann-Whitney U test; Fig. 2b,c)]. In addition, we observed protection of the SGCs in the apical turn by Ex and CR (Fig. 2d,e), consistent with the histological changes in OHCs [SGCs in basal turn: Ex, p = 0.61; CR, p = 0.83) (SGCs in apical turn: Ex, p < 0.01; CR, p < 0.001) (n = 4 mice per group; Mann-Whitney U test)].

In order quantify the histological results, we evaluated the rescue effect that Ex and CR exerts on OHC and SGC. First, we compared the changes in OHC survival rates and SGC densities between mice in the Pre group mice and those in the control, Ex and CR group mice at 10 months. The OHC reduction rate was 49.0% in control mice, 22.0% in Ex, and 18.8% in CR (data are averages in the apical turn; Fig. 2c; see Materials and Methods for more details). The SGC reduction rate was 39.2% in control mice, 15.6% in Ex, and 20.5% in CR (data are averages in the apical turn; Fig. 2f). Ex and CR rescue effects on OHC are indicated by the differences in the OHC reduction rate between control and Ex mice or control and CR mice, while those on the SGC are indicated by the differences in the SGC reduction rate between control and Ex mice or control and CR mice. The results showed that the Ex and CR rescue effects on OHC were 27.0% and 30.1%, respectively (Fig. 2c), while the Ex and CR rescue effects on SGC were 23.6%, and 18.6%, respectively (Fig. 2f). Although OHC survival rates and SGC densities in Ex and CR mice did not return to those of the Pre group mice (Ex: OHC survival, p < 0.01; SGC density, p < 0.01; CR: OHC survival, p < 0.01; SGC density, p < 0.001; n = 4 mice per group; Mann-Whitney U test; Fig. 2c,e), the results indicate that Ex and CR can reduce degenerative changes in the OHCs and SGCs in the apical turn.

The percentages of anti-8-hydroxy-2′-deoxyguanosine (8-OHdG)-positive SGC cells (a marker of oxidative stress) in Ex and CR mice were significantly lower than those in control mice [8-OHdG in the basal turn: Ex, p = 0.12; CR, p = 0.8) (8-OHdG in the apical turn: Ex, p < 0.001; CR, p < 0.001) (n = 3 mice per group; Mann-Whitney U test; Fig. 2g,h)]. These results indicate that Ex and CR reduced the age-related degeneration of the OHC and the SGC and reduced oxidative stress in the SGC in the apical regions of the cochlea. Thus, Ex and CR induced positive effects in the auditory system, consistent with previous results^2,10^.

### Ex and CR induce a reduction in the number of OSNs in the DM region of the OE

**Figure 3:**
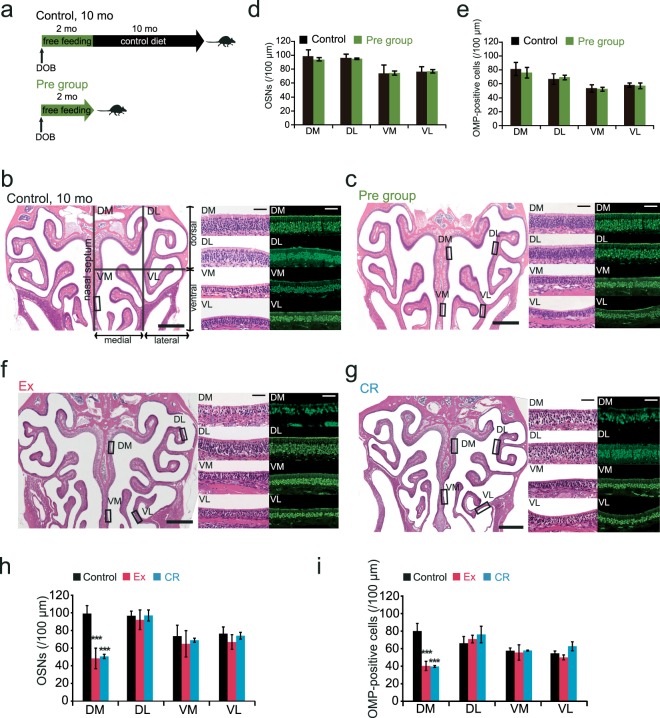
Long-term Ex and CR induce a reduction in the number of OSNs in the dorsomedial region of the olfactory epithelium. **(a)** Time course of the experimental design. Control group: mice were presented with food ad libitum for the first 2 mo of life and were then fed the control diet for a further 10 mo before tissue fixation. Pre group: mice were presented with food ad libitum for the first 2 mo of life and then euthanized for tissue fixation. **(b,c)** Photomicrographs of representative coronal sections of the olfactory epithelium (OE) in control (B) and Pre mice (C). Left images, lower magnification; middle and right images, higher magnification of the selected rectangular regions with hematoxylin and eosin staining (middle) and anti-OMP staining (right). The unilateral OE was divided into four areas: dorsomedial (DM), dorsolateral (DL), ventromedial (VM), and ventrolateral (VL). Scale bars: 300 µm at low magnification, 50 µm at higher magnification. **(d,e)** Number of OSNs (D) and OMP-positive cells (E) in each area. There were no significant differences between control and Pre mice in any area (Mann-Whitney U test). **(f**,**g)** Photomicrographs of representative coronal sections in Ex and CR mice. Left images, lower magnification; middle and right images, higher magnification of the selected rectangular regions with hematoxylin and eosin staining (middle) and anti-OMP staining (right). Scale bars: 300 µm at low magnification, 50 µm at higher magnification. **(h,i**) Number of OSNs (H) and OMP-positive cells (I) in each area (DM, DL, VM, and VL) in Ex and CR mice. There were significant changes in the DM area in Ex and CR mice compared with control mice (**p < 0.01; Mann-Whitney U test).

We compared coronal sections of the OE from 12-month-old control mice with those of 2-month-old mice that had not undergone Ex or CR (the Pre group; Fig. 3a–c). The number of OSNs differed among the different regions of the OE (DM, DL, VM, and VL; see Materials and Methods) in both the Pre group and control mice. The number of anti-olfactory marker protein (OMP)-positive cells was similar between control and Pre group mice [(OSNs: DM, p = 0.41; DL, p = 0.64; VM, p = 0.95; VL, p = 0.59) (OMP: DM, p = 0.61; DL, p = 0.63; VM, p = 0.78; VL, p = 0.45) (n = 3 mice per group; Mann-Whitney U test; Fig. 3d,e)]. These results indicate that 10 months of the control diet did not induce any histological changes in the OE.

Next, we examined whether 10 months of Ex or CR affects the number of OSNs in the OE (Fig. 3f–g). The number of OSNs and OMP-positive cells in the DM region was significantly lower in Ex and CR mice than in age-matched controls. In the DL, VM, and VL regions, however, we did not observe significant differences between the experimental and control groups [OSNs: (Ex: DM, p < 0.001; DL, p = 0.57; VM, p = 0.08; VL, p = 0.09) (CR: DM, p < 0.001; DL, p = 0.34; VM, p = 0.17; VL, p = 0.8), OMP: (Ex: DM, p < 0.001; DL, p = 0.2; VM, p = 0.31; VL, p = 0.14) (CR: DM, p < 0.001; DL, p = 0.39; VM, p = 0.39; VL, p = 0.42) (n = 3 mice per group; Mann-Whitney U test; Fig. 3h,i)]. These results indicate that Ex and CR induce histological changes selectively in the DM region of the OE.

The OE is subdivided into dorsal and ventral zones based on the differential expression of specific types of odorant receptors and intracellular molecules^12^. The dorsal zone is distributed in the DM region of the OE, where OSNs express NADPH quinone oxido-reductase 1 (NQO1)^13^. Therefore, we hypothesized that Ex and CR would induce histological changes selectively in the dorsal zone and affect NQO1-expressing cells.

**Figure 4:**
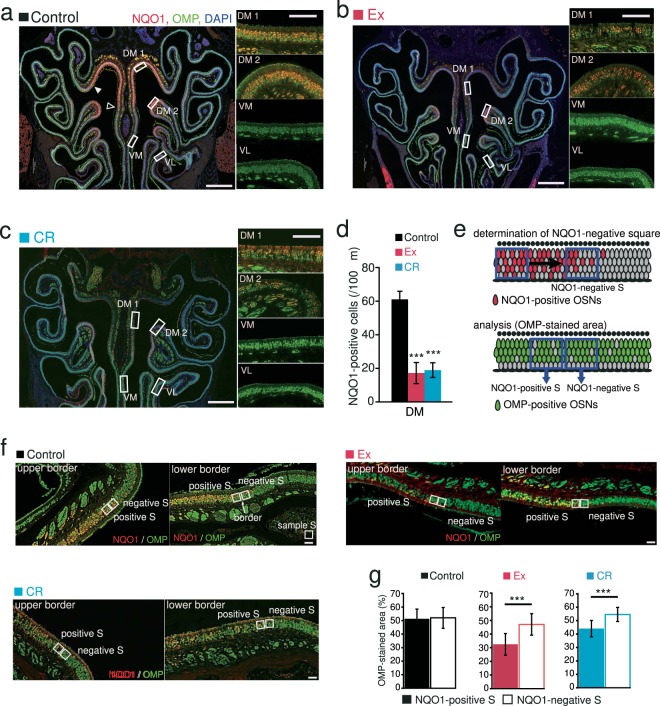
Ex- and CR-induced reduction in OSN numbers occur selectively in the NQO1-positive dorsal zone of the olfactory epithelium. **(a–c)** Photomicrographs of representative coronal sections in control, Ex and CR mice. The rectangular portions of the OE in the left-hand photographs are enlarged in the right-hand images (red, anti-NQO1; green, anti-OMP; blue, DAPI). DM1, upper nasal septum in the dorsomedial area; DM2, upper concha bullosa in the dorsomedial area. Closed triangle, upper border of the NQO1-positive area; open triangle, lower border of the NQO1-positive area. Scale bars: 300 µm at low magnification, 50 µm at higher magnification. **(d)** The number of NQO1-positive cells in the dorsal zone in control, Ex and CR mice. The number of NQO1-positive cells was significantly lower in Ex and CR mice than in control mice (***p < 0.001; Mann-Whitney U test). **(e)** Schematic diagram for the determination of the NQO1-positive and -negative squares. Upper, the NQO1-negative square was defined as the 30 mm square closest to the border with a staining intensity <2 SD below the mean intensity measured in the NQO1-positive area of the OE. The NQO1-positive square (positive S) was defined as the 30 mm square adjacent to the NQO1-negative square. The intensity of OMP staining in NQO1-positive and -negative squares was compared. **(f)** Photomicrographs of the upper (closed triangle in a) and lower borders (open triangle in a) in control, Ex and CR mice (red, anti-NQO1; green, anti-OMP). The borders between NQO1-positive and -negative areas are shown (arrow). Scale bar, 30 µm. **(g)** Comparison of OMP-immunostained areas in NQO1-positive and -negative squares in control, Ex and CR mice. The OMP-stained area in the NQO1-positive square was smaller than that in the NQO1-negative square in Ex and CR mice, but not in control mice (***p < 0.001; Mann-Whitney U test).

We examined whether the DM region of the OE that showed histological changes in response to Ex and CR corresponds to the dorsal zone. NQO1-positive OSNs were observed in the upper nasal septum (DM1) and upper concha bullosa (DM2; Fig. 4a). NQO1-positive OSNs were not observed in other areas (Fig. 4a). The number of NQO1-positive cells in the dorsal zone (DM1 and DM2) was significantly lower in Ex and CR mice than in age-matched control mice (Ex, p < 0.001; CR, p < 0.001; n = 3 mice per group; Mann-Whitney U test; Fig. 4b–d).

To further examine histological changes at the border between NQO1-positive and -negative areas, we compared OMP immunoreactivity in NQO1-negative (negative S) and NQO1-positive (positive S) squares (Fig. 4e,f). OMP immunoreactivity was significantly lower in the NQO1-positive square than in the NQO1-negative square in Ex and CR mice (control, p = 0.75; Ex, p < 0.001; CR, p < 0.001; n = 3 mice per group; Mann-Whitney U test; Fig. 4g). To exclude the possibility that differences in exposure time of OMP staining had a greater influence on the assessment of OMP immunoreactive areas in the NQO1-negative square of Ex and CR than in control mice, we compared OMP-immunoreactive areas in the NQO1-negative square among control, Ex, and CR. The results showed no significant differences of the OMP-immunoreactive area in NQO1-negative square among control, Ex, and CR (control vs. Ex in NQO1-negative square, P = 0.11; control vs. CR in NQO1-negative square, P = 0.49, Mann-Whitney U test; Fig. 4g), indicating that the difference in the OMP-immunoreactive area was not due to a difference in exposure time of OMP-staining. Taken together, these results indicate that 10 months of Ex or CR can induce damage in the dorsal zone of the OE.

**Figure 5:**
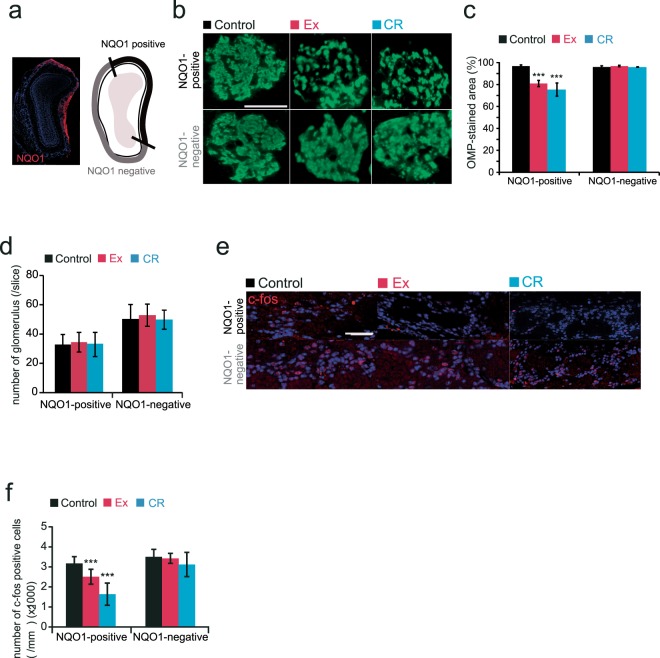
Ex and CR reduce glomerular size and the neuronal response to odorants selectively in the dorsal domain of the olfactory bulb. **(a)** Area of analysis in the olfactory bulb (OB). Left, coronal section of the OB stained with anti-NQO1 antibody (red) and DAPI (blue). Right, schematic diagram of the OB showing NQO1-positive and -negative areas. **(b)** Representative coronal sections stained with anti-OMP (green) in control, Ex and CR mice. Each circled area corresponds to a glomerulus. Scale bar, 50 µm. **(c)** Summary of the ratio of areas stained and unstained with OMP. The OMP-stained area of NQO1-positive OB was significantly smaller in Ex and CR mice than in control mice (***p < 0.001; Mann-Whitney U test). There were no differences in the OMP-stained area between groups in the NQO1-negative OB (Mann-Whitney U test). **(d)** The number of glomerulus in control, Ex and CR mice in NQO1-positive and -negative OB. There were no significant differences in the number of glomeruli between groups in NQO1-positive and -negative OB (Mann-Whitney U test). **(e)** Representative OB coronal sections stained with anti-c-fos antibody for the NQO1-positive and NQO1-negative OB in control, Ex and CR mice. Scale bar, 50 µm. **(f)** The number of c-fos-positive cells in control, Ex and CR mice in NQO1-positive and -negative OB. The density of c-fos-positive cells in the NQO1-positive OB in Ex and CR mice was lower than that in control mice. There were no differences between groups in the density of c-fos-positive cells in NQO1-negative OB (***p < 0.001; Mann-Whitney U test).

To examine the effect of Ex and CR on axonal projections to the glomeruli in the OB, we measured the size of the OMP-stained area within individual glomeruli of the OB in Ex and CR mice. The axonal target glomeruli were selected from the NQO1-positive and -negative areas of the OB (Fig. 5a). We observed the size of the OMP-stained area in NQO1-positive OB in Ex and CR mice was smaller than that in age-matched control mice (Fig. 5b,c). We did not detect any differences in the NQO1-negative OB [(NQO1-positive: Ex, p < 0.001; CR, p < 0.001) (NQO1-negative: Ex, p = 0.63; CR, p = 0.2) (n = 3 mice per group; Mann-Whitney U test; Fig. 5b,c)]. We conducted an additional analysis, in which we compared the number of glomeruli per slice (21 slices, 3 mice) in NQO1-positive and NQO1-negative OBs, in order to determine whether the effect of decreased OMP density was solely due to their contribution to glomeruli or due to a decrease in the total NQO1-positive area in the OB. The results showed that the number of NQO-1-positive glomeruli did not differ significantly among control, Ex, and CR mice (NQO1-positive: Ex, p = 0.56; CR, p = 0.85; Mann-Whitney U test). In addition, the number of NQO-1 negative glomeruli did not differ significantly among control, Ex, and CR mice (NQO1-negative: Ex, p = 0.65; CR, p = 0.52; Mann-Whitney U test, Fig. 5d). These results indicate that the experimental interventions resulted in the projection of fewer axons from OSNs in the dorsal zone.

To examine whether dorsal-zone-specific injury to the OE disrupts the function of stable sensory inputs to the glomeruli, we measured the expression of the neural activity marker c-fos within the glomerular layer in NQO1-positive and -negative OB in Ex and CR mice after odor stimulation. Stimulus odorants (aldehydes, lactones, and esters) induced c-fos expression within the glomerular and granule cell layers broadly throughout the OB. We focused our analysis on glomerulus region-specific c-fos expression within the glomerular layer because the increase in c-fos expression within the glomerular layer is glomerulus region-specific, and depends on afferent inputs from OSNs, while c-fos expression in the granule cell layer is also affected by centrifugal inputs^14^. If relatively weak afferent inputs from NQO1-positive OE to the NQO1-positive OB are observed, fewer c-fos-positive cells would be expected within the glomerular layer of the NQO1-positive OB than in the NQO1-negative OB.

We observed a smaller reduction in the number of c-fos-positive cells within the NQO1-positive glomerular layer of the OB in Ex and CR mice than in age-matched control mice (Fig. 5e,f). We did not detect any significant differences in the number of c-fos-positive cells within the NQO1-negative glomerular layer of the OB in Ex or CR mice relative to their age-matched controls [(NQO1-positive: Ex, p < 0.001; CR, p < 0.001) (NQO1-negative: Ex, p = 0.72; CR, p = 0.1) (n = 3 mice in Ex, n = 4 mice in CR; Mann-Whitney U test; Fig. 5e,f)]. These results suggest that the decrease in dorsal-zone OMP-immunoreactive cells, OSNs, and their axonal projections to the glomeruli is associated with a decrease in the glomerular response to odorants.

### Long-term Ex or CR is required for dorsal-zone-specific injury

**Figure 6:**
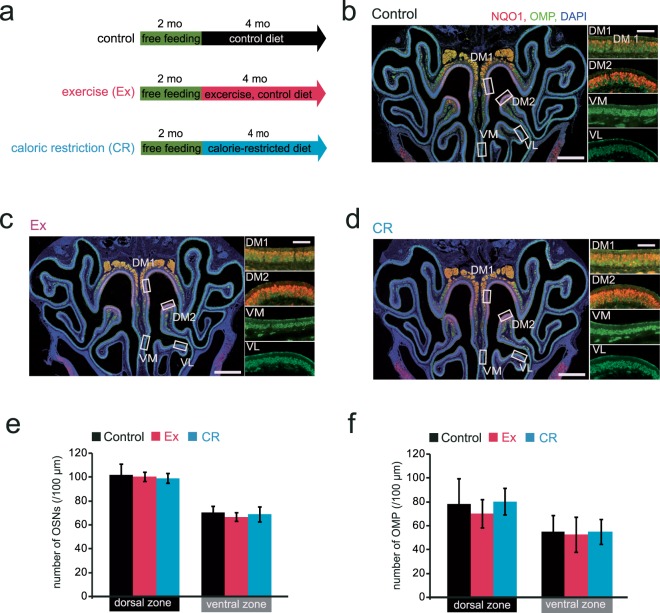
Short-term Ex and CR did not induce a reduction in OSN numbers in the dorsal zone of the olfactory epithelium. **(a)** Time course of the experimental design. At 2 mo, mice were divided into three groups: control, 4 mo with the control diet; Ex, 4 mo running-wheel exercise with the control diet; CR, 4 mo with the CR diet. **(b–d)** Photomicrographs of representative coronal sections of the OE in control, Ex and CR mice. The rectangular portions of the OE in the left-hand photographs are enlarged in the right-hand images. Scale bars: 300 µm at low magnification, 50 µm at higher magnification. **(e,f)** Number of OSNs (e) and OMP-positive cells (f) in the dorsal and ventral zones. There were no significant histological changes between control, Ex and CR mice in either zone (Mann-Whitney U test).

Previous reports indicated that 4–6 weeks of CR in mice improves glucose tolerance, insulin sensitivity, and inflammatory responses in the brain^15,16^. These observations imply that short-term CR exposure of a few weeks may induce energy metabolic alterations in systemic levels, and may result in histological changes in the OE. To examine whether short-term Ex or CR induces dorsal-zone-specific injury in the OE, we assessed histological changes in the OE after 4 months of Ex or CR (Fig. 6a). We did not observe significant differences in the number of OSNs or OMP-positive cells in any of the four areas of the OE between Ex and CR mice and age-matched control mice [(OSNs in the dorsal zone: Ex, p = 0.61; CR, p = 0.16) (OSNs in the ventral zone: Ex, p = 0.08; CR, p = 0.61) (OMP in the dorsal zone: Ex, p = 0.36; CR, p = 0.62) (OMP in the ventral zone: Ex, p = 0.64; CR, p = 0.87) (n = 3 mice per group; Mann-Whitney U test; Fig. 6b–f)]. These results indicate that 4 months of Ex or CR does not induce histological changes in the dorsal zone of the OE.

### Ex and CR increase proliferation and apoptosis in OSNs

**Figure 7:**
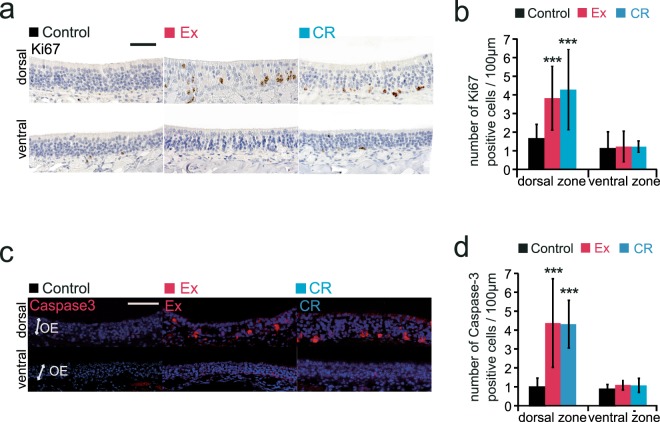
Ex and CR increase the numbers of Ki67-positive cells and apoptotic cells in the dorsal zone of the olfactory epithelium. **(a)** Representative images of Ki67-positive cells in the dorsal and ventral zones in control, Ex and CR mice. Scale bar, 50 μm. **(b)** The number of Ki67-positive cells in dorsal and ventral zones in control, Ex and CR mice. In the dorsal zone, the number of Ki67-positive cells was significantly higher in Ex and CR mice than in control mice (***p < 0.001; Mann-Whitney U test), whereas there was no significant difference in the number of Ki67-positive cells in the ventral zone between Ex and CR mice and the controls. **(c)** Representative images of caspase-3-positive cells in dorsal and ventral zones in control, Ex and CR mice. Scale bar, 50 μm. **(d)** The number of caspase-3-positive cells in dorsal and ventral zones in control, Ex and CR mice. In the dorsal zone, the number of caspase-3-positive cells was significantly higher in Ex and CR mice than in control mice (***p < 0.001; Mann-Whitney U test). There was no significant difference in the number of caspase-3-positive cells in the ventral zone between Ex and CR mice and the controls.

Fewer OSNs in the dorsal zone may result from decreased OSN proliferation, increased cell death, or both. To examine the mechanism underlying the reduction in OSNs in the dorsal zone of the OE following Ex and CR, we examined the number of OE cells that were positive for Ki67 (a marker of proliferation) and caspase-3 (a marker of cell death). The number of dorsal-zone Ki67-positive cells was significantly higher in Ex and CR mice than in age-matched controls (Fig. 7a). However, there were no significant increases in Ki67-positive cells in the ventral zone [(dorsal: Ex, p < 0.001; CR, p < 0.001) (ventral: Ex, p = 0.73; CR, p = 0.56) (n = 3 mice in Ex, 4 mice in CR; Mann-Whitney U test; Fig. 7b)]. We also found more caspase-3-positive cells in the dorsal zone in Ex and CR mice than in their age-matched controls, but no significant differences in caspase-3-positive cells in the ventral zone [(dorsal: Ex, p < 0.001; CR, p < 0.001) (ventral: Ex, p = 0.09; CR, p = 0.16) (n = 3 mice in Ex, 4 mice in CR; Mann-Whitney U test; Fig. 7c,d)]. These results indicate that Ex and CR increase the mitotic rate of progenitor basal cells, but that the recovery of cell numbers was incomplete because of the increase in apoptotic OSNs in the dorsal zone.

### Oxidative stress in dorsal-zone OSNs is strongly linked to NQO1 activity

**Figure 8:**
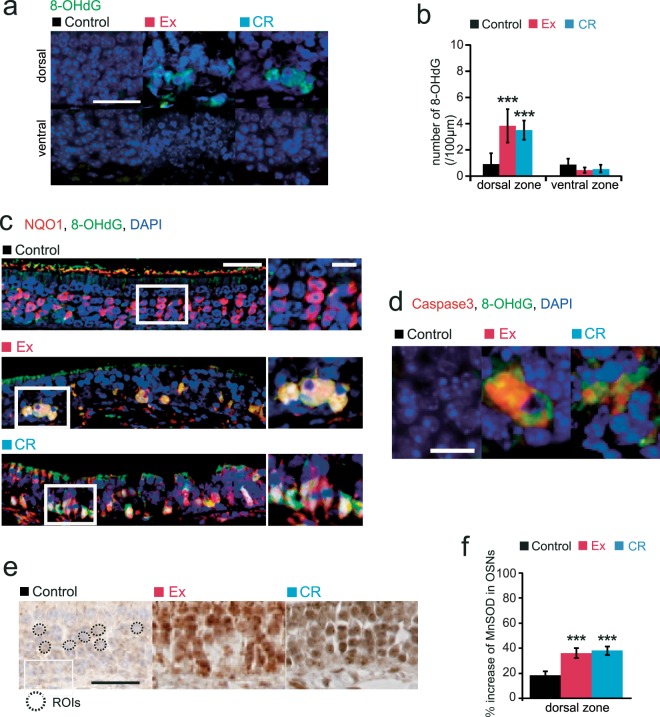
Ex and CR increase the numbers of 8-OHdG-positive cells in the dorsal zone of the olfactory epithelium. **(a)** Representative images of 8-OHdG-positive cells in the dorsal zone in control, Ex and CR mice. Scale bar, 50 μm. **(b)** The number of 8-OHdG-positive cells in the dorsal zone in control, Ex and CR mice. The number of 8-OHdG-positive cells was significantly higher in Ex and CR mice than in control mice (***p < 0.001; Mann-Whitney U test). **(c)** Representative images anti-8-OHdG (green) and anti-NQO1 (red) staining in control, Ex and CR mice. The rectangular portions of the OE in the left-hand photographs are enlarged in the right-hand images. A significant number of 8-OHdG-positive cells were co-stained with anti-NQO1. Scale bars: 30 µm at low magnification, 10 µm at higher magnification. **(d)** Representative images stained with anti-8-OHdG (green) and anti-caspase-3 (Casp3, red) in control, Ex and CR mice. A significant number of 8-OHdG-positive cells were co-stained with anti-casp3. Scale bar, 20 µm. **(e)** Representative images of anti-MnSOD staining in control, Ex and CR mice showing the intensity of anti-MnSOD immunostaining in the OSNs (dotted circles), and axon bundles, and the intensity of immunostaining in OSNs compared with that in the control area. Scale bar, 20 µm. **(f)** MnSOD immunostaining in the OSNs of axon bundles of control, Ex and CR mice. Relative intensity of staining was significantly higher in the OSNs of Ex and CR mice than in those of control mice (***p < 0.001; Mann-Whitney U test).

NQO1, a cytosolic flavoenzyme, protects against oxidative stress through the removal of quinones and other xenobiotic metabolic products^9^. However, NQO1 also mediates ROS generation via conjugation with superoxide dismutase (SOD), leading to enhanced oxidative stress^9^. We hypothesized that increased ROS generation, mediated by NQO1 activity, would induce oxidative stress, resulting in increased apoptosis in the dorsal zone of the OE. We examined the expression of 8-OHdG in Ex and CR mice as a marker of oxidative stress. We observed significantly more 8-OHdG-positive cells in the dorsal zone in Ex and CR mice than in the age-matched controls, but no increase of 8-OHdG-positive cells in the ventral zone [(8-OHdG in the dorsal zone: Ex, p < 0.001; CR, p < 0.001) (8-OHdG in the ventral zone: Ex, p = 0.09; CR, p = 0.16) (n = 3 mice per group; Mann-Whitney U test; Fig. 8a,b)]. Furthermore, we co-labeled OSNs with anti-NQO1 and anti-8-OHdG to examine the role of oxidative stress in NQO1 expression. A significant number of 8-OHdG-positive cells also expressed NQO1 immunoreactivity (Ex, 202 of 222 8-OHdG-positive cells analyzed, 91.0%; CR, 149 of 163, 91.4%; n = 3 mice per group; Fig. 8c). These results indicated that NQO1-positive cells had increased oxidative stress.

To examine whether increased oxidative stress in the dorsal zone could induce apoptosis of OSNs, we co-labeled OSNs with anti-caspase-3 and anti-8-OHdG. We found that a significant number of 8-OHdG-positive cells also expressed anti-caspase-3 immunoreactivity (Ex, 245 of 277 8-OHdG-positive cells analyzed, 88.4%; CR, 187 of 214, 87.4%; n = 3 mice per group; Fig. 8d). These results indicate that increased oxidative stress induces apoptosis of the OSNs in the dorsal zone in Ex and CR mice.

According to our hypothesis, NQO1 mediates ROS generation by increasing MnSOD activity, leading to enhanced oxidative stress in the dorsal zone. We measured the intensity of anti-MnSOD immunoreactivity to quantify MnSOD activity (dotted circles, Fig. 8e). We compared the relative intensity of immunostaining between OSNs and axon bundles in the dorsal zone. The results revealed that staining intensity was higher in Ex and CR mice than in age-matched control mice (Ex, p < 0.001; CR, p < 0.001; n = 3 mice per group; Mann-Whitney U test; Fig. 8f), indicating that enhanced ROS generation in Ex and CR mice was involved in the bioactivation of NQO1 by high MnSOD activity and led to prominent oxidative stress in the dorsal zone.

## Discussion

Ex and CR have been reported to promote longevity and metabolic health in mammalian species and to reduce age-related degenerative changes in individual organs^5,6,17^. In mice and humans, age-related hearing loss (AHL) affects the high-frequency (basal) region of the cochlea more profoundly than the low-frequency (apical) region^2^. Long-term voluntary Ex significantly delays AHL progression at low (8 kHz) and middle (16 kHz) frequencies in aged mice^10^. CR suppresses apoptotic cell death in the cochlea and prevents late-onset AHL in mice^11,18^. Consistent with these previous reports, we observed that the Ex and CR regimens reduced age-related degenerative changes in OHCs and SGC in the apical turn of the cochlea. By contrast, Ex and CR had negative functional and structural effects in the olfactory system. We observed more apoptosis and proliferation in the dorsal zone of the OE in Ex and CR mice than in control mice. This is similar to what was observed in the injured OE. For example, methimazole, an olfactotoxic drug, disrupts existing OSNs throughout the OE by activating the apoptotic cascade in OSNs^19^. Injury-induced loss of OSNs causes a prompt and massive regeneration of new OSNs through the proliferation of progenitor cells, and eventually the OE returns to its pre-injury level by 1–2 months^20,21^. However, when the OE is exposed continuously to pathogens or toxins, the apoptosis of OSNs is increased, resulting in OSN loss irrespective of the increased mitosis of progenitor basal cells.

In the analysis of caspase 3 and Ki67-positive cells, we could not directly compare the numbers of caspase 3- and Ki67-positive cells in a histological section to estimate increases or decreases in their cell numbers. The ability to visualize dying cells using histological techniques depends on the length of time between cell death and phagocytosis. There is evidence showing that the histologically visible stages of the apoptotic process are very short, lasting from a few minutes to a maximum of 3 hr^22^. Accordingly only limited numbers of apoptotic cells could be detected in a histological section at any one time. Contrary to the very short detectable time of dying cells, the detection of mitotic proteins such as Ki67 may be possible over a longer time interval^23^. Because of the difference in the time interval between the detection of caspase 3 and Ki67, the extent of cell number increase or decrease over long time intervals cannot be reliably inferred from the numbers of cells in a histological section at any one time. As a possible explanation for decreased numbers of OSNs in the dorsal zones of Ex and CR mice, we speculate that, during the long time intervals, the increase in the total number of apoptotic OSNs was greater than the increase in new OSNs generated by the increased mitosis of progenitor basal cells (Fig. 7). It is possible that this imbalance between neuron regeneration and death results in the incomplete replacement of OSNs in the dorsal zone.

Voluntary long-term Ex and CR can induce changes in the AMPK, SIRT1, mTOR, GH/insulin/IGF1, and FGF21 pathways. These pathways respond to cellular bioenergetics or to sugars^6,24,25^. We predicted that Ex and CR would act to reduce anabolic responses and oxidative stress. However, we observed a significant increase in immunoreactivity for 8-OHdG, a marker of oxidative stress, in the dorsal zone of the OE, but not in the ventral zone, suggesting that different cellular mechanisms regulate the generation of oxidative stress in the dorsal and ventral zones of the OE. The preferential generation of oxidative stress in the dorsal zone may be associated with NQO1 activity conjugated with MnSOD, because we found significant co-localization of oxidative stress with NQO1 expression and markers of apoptosis.

NQO1, a cytosolic flavoenzyme, catalyzes the 2-electron reduction of quinones and aromatic nitro compounds and protects cells against quinone toxicity. However, NQO1 bioactivation within metabolic pathways involving multiple enzymes is not simple. In particular, the conjugation of NQO1 with MnSOD facilitates the generation of ROS through the reaction of unstable hydroquinones with oxygen, leading to oxidative stress^9,26^. We hypothesize that the physiological properties of NQO1 in the presence of high MnSOD activity may cause OSNs to become highly susceptible to damage from environmental quinone agents or endogenous neurotoxins. MnSOD does have antioxidant enzymatic properties, but an imbalance between ROS generation and antioxidant defenses within the dorsal zone might induce continuous oxidative damage. The oxidative damage would result in structural and functional damage to dorsal-zone OSNs despite the generation of new neurons. Pathological conditions, such as that caused by the intraperitoneal injection of olfactory toxins or the inhalation of hydrogen sulfide, can induce localized neural degeneration of OSNs in the dorsal zone^27^. Together, these data suggest that NQO1 activity increases OSN susceptibility to the harmful action of other substrates.

Differential susceptibility to damage in each zone may be associated with different airflows in the nasal cavity. It has been suggested that the dorsal–medial OE is subjected to extensive ventilation during breathing^28^. Ex increases airflow, which may result in increased OSN exposure to various neurotoxins. However, the demarcation between high and low airflow is not well defined, in contrast to the sharp demarcation between the healthy (ventral zone) and damaged (dorsal zone) OE. Consequently, we do not believe different airflows in the nasal cavity induced the dorsal-zone-specific damage observed in this study.

Other possible mechanisms of dorsal-zone-specific damage include alterations in internal hormonal signaling, because nutritional status impacts olfactory sensitivity^8,29^. The OE and OB express high levels of mRNAs for anorexia-signaling hormone receptors, such as leptin, insulin, and IGF1^29^. Metabolic hormones can affect neuron survival and their activity in olfactory networks when targeting their receptors^29,30^. Insulin can increase the number of cultured OSNs *in vitro* and prevents the apoptosis of OSNs via activation of intracellular cAMP in adult rats after injury^30^. In this study, Ex and CR may have caused a decrease in systemic insulin/IGF1 signaling, thereby affecting the regulation of cell dynamics in the OE. In addition to alterations in internal hormonal signaling, calorie-restricted animals show reduced natural killer cell function and reduced ability to combat virus infection effectively^31,32^. We hypothesize that the high susceptibility of dorsal-zone OSNs to damage is facilitated by a decrease in insulin/IGF1 signaling and high chance of infection.

**Figure 9:**
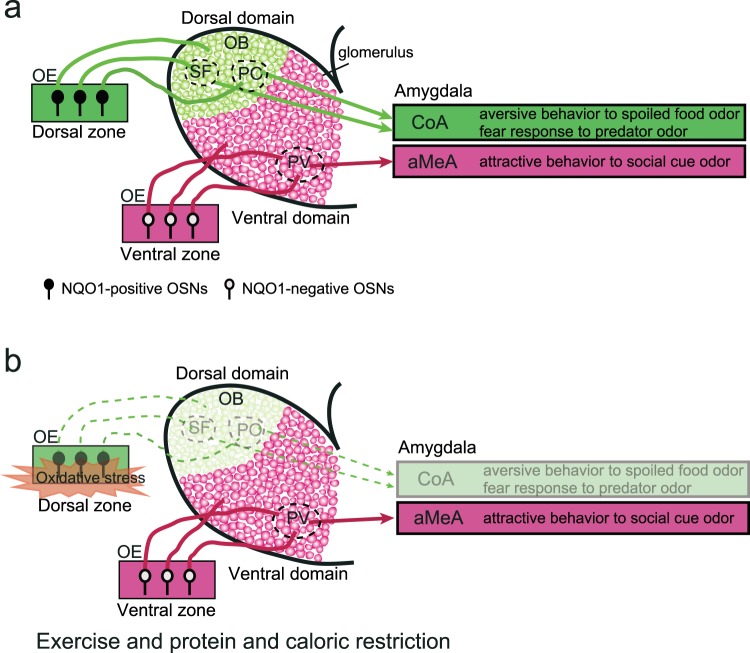
Schematic diagram illustrating two parallel olfactory pathways. **(a)** NQO1-positive OSNs in the dorsal zone of the OE project to the dorsal domain in the main OB. Aversive odor signals are transmitted from the dorsal domain in the main OB (spoiled-food-odor-responsive glomeruli, SF; predator-odor-responsive glomeruli, PO) to the cortical amygdala (CoA), mediating the aversive behavior to spoiled food odors and the fear response to predator odors. NQO1-negative OSNs in the ventral zone project to the ventral domain in the main OB. Attractive social odor signals are transmitted from the posteroventral (PV) part of the main OB to the anterior amygdala (aMeA), mediating attractive behavior to social odor cues. OE, olfactory epithelium; OB, olfactory bulb; OSNs, olfactory sensory neurons. **(b)** Long-term Ex and CR reduce the number of functional OSNs in the dorsal zone that give rise to the dorsal olfactory pathway. By contrast, long-term Ex and CR have little effect on OSNs in the ventral zone that give rise to the ventral pathway.

OSNs in the dorsal zone project to the dorsal domain in the main OB. Many mitral cells in the dorsal domain project to the cortical amygdala and mediate aversive behavior to spoiled odors and fear responses to predator odors (dorsal domain stream, Fig. 9a). OSNs in the ventral zone project to the ventral domain in the main OB^33^. Mitral cells located in the posteroventral part of the ventral domain project to the anterior medial amygdala, and mediate attractive social behaviors such as mating and conspecific social odor recognition (ventral domain stream, Fig. 9a)^34^. Long-term Ex and CR had little effect on OSNs in the ventral zone, suggesting that these interventions would not change the odor threshold for the induction of positively motivated behaviors, such as attractive social behaviors (Fig. 9b). By contrast, selective reduction of OSNs in the dorsal zone under long-term Ex and CR interventions is expected to decrease sensitivity to odors that activate the dorsal domain stream, and thus increase the odor threshold for the induction of negatively motivated behaviors, such as aversive behavior and fear responses (Fig. 9b). In wild animals, high sensitivity and subsequent prompt reactions to danger-signaling odors from spoiled food and predators are critical for survival and adaptation to the external environment. Accordingly, this critical trade-off between the prevention of age-related degenerative changes in the auditory system and structural and functional defects of the dorsal domain stream in the olfactory system may adversely affect species perpetuation.

In future studies, it will be necessary to determine the amount of exercise and caloric restriction that maximizes metabolic wealth and minimizes frailty in the olfactory system, and to explore whether the same interventions result in similar effects in humans.

## Materials and Methods

### Animals

Individually caged male C57BL/6J mice were used. All mice were maintained on a standard purified mouse diet for 2 months prior to the start of the experiment. At 2 months of age, mice were allocated randomly into three groups: control, voluntary wheel running Ex, and CR. Animals in each group were fed different diets according to their treatment condition (Table 1). Animals in the Pre group, employed to control for the effects of the control diet were fed ad libitum until 2 months of age and then sacrificed for histology.

**Table 1:** Nutrient composition of the diets used in this study.

Nutrient composition	Control, Ex	CR
Casein	14.0%	14.0%
β-Cornstarch	46.6%	26.1%
α-Cornstarch	15.5%	17.4%
Sucrose	10.0%	10.0%
Soybean oil	4.0%	4.0%
Cellulose powder	5.0%	23.6%
AIN-93G mineral mixture	3.5%	3.5%
AIN-93 vitamin mixture	1.0%	1.0%
Cholinebitartrate	0.3%	0.3%
Tertiary butyl hydroquinone	0.1%	0.1%
Total	100.0%	100.0%
Total calorie (Kcal/100 g)	353.6	280.9

Control and exercise (Ex) mice were fed a diet of 353.6 Kcal·100 g^−1^. Caloric restriction (CR) mice were fed a diet of 280.9 Kcal·100 g^−1^, with 20% less cornstarch than AIN-93M and more cellulose.

### Caloric restriction

Two custom diets were developed based on AIN-93M^35^ and manufactured by a local stock-feed company (Oriental Yeast Co., Tokyo, Japan). All food was presented as dry pellets, with the amount calculated per gram by electronic scales every 2 days according to the daily food intake data (approximately 4 g per day). The pellets used in the custom diets varied only in their carbohydrate ratios (Table 1). Control and Ex mice were fed a diet of 353.6 Kcal·100 g^−1^. CR mice were fed a diet of 280.9 Kcal·100 g^−1^, with 20% less cornstarch than AIN-93M and more cellulose.

### Voluntary running wheel

The voluntary wheel-running apparatus consisted of a plastic slatted wheel attached to a slanted central rod planted into a support base that houses batteries (wheel diameter, 15.5 cm; Med Associates Inc., St Albans, VT, USA). The entire unit fits into most standard mouse cages. When the mouse rotates the wheel, an electronic signal is transmitted wirelessly to a hub. The number of revolutions per minute was recorded by Wheel Manager software (Med Associates Inc.). Activity was recorded as the distance traveled (km) per month. Data were exported to a spreadsheet for further analysis.

### Counting hair and spiral ganglion cells

The presence of cochlear hair cells (HCs) was recorded if the cell body and cuticular plate remained intact. The number of HCs at the basal and apical turns was counted in at least ten sections per animal. HC (IHC) and outer HC (OHC) survival rates were calculated using the following formulae: IHC survival rate (%) = 100 × [(number of examined specimens with IHCs present)/number of examined specimens]; OHC survival rate (%) = 100 × [(number of examined specimens with OHCs present)/number of examined specimens].

Spiral ganglion cell (SGC) density (number·mm^−2^) at each turn was calculated per animal by dividing the number of SGCs by the area of Rosenthal’s canal (Image J, National Institutes of Health; NIH). SGC survival rates were calculated using the following formula: SGC survival rate (%) = 100 × [(number of examined SGC density)/number of examined SGC density in pre group].

The OHC reduction rate was calculated using the following formula: the OHC survival rate in pre-group mice - the OHC survival rate in control, Ex, and CR mice. The SGC reduction rate was calculated using the following formula: the SGC survival rate in pre group mice - the SGC survival rate in control, Ex, and CR mice.

The percentage of the 8-OHdG positive cells was calculated at each turn by dividing the number of the 8-OHdG-positive SGC cells by the total number of SGC cells (number of 8-OHdG-positive SGC cells/total number of SGC cells × 100).

### Immunohistochemistry

Mice were perfused with 4% paraformaldehyde in 0.1% phosphate buffer and post-fixed for 24 h in the same fixative. The head tissue, including the cochlea, OE, and olfactory bulb (OB), was decalcified with 10% EDTA solution, pH 7.0, and embedded in paraffin. Coronal sections were cut at 4 μm thickness and mounted on silane-coated slides. Deparaffinized sections were autoclaved at 121 °C for 20 min in Target Retrieval Solution (S1700; Dako) for antigen retrieval. Immunohistochemistry was performed with the following antibodies: anti-olfactory marker protein (OMP, goat polyclonal, 1:2000 dilution; Wako Chemicals), anti-NQO1 (rabbit polyclonal, 1:300; Cell Signaling Technology), anti-activated caspase-3 (rabbit polyclonal, 1:300; Cell Signaling Technology), anti-Ki67 (mouse monoclonal, 1:300; BD Biosciences), anti-c-fos (rabbit polyclonal, 1:50; Santa Cruz Biotechnology), anti-MnSOD (rabbit monoclonal, 1:100; Epitomics Inc.), and anti-8-hydroxy-2′-deoxyguanosine (8-OHdG, goat polyclonal antibody, 1:100; Alpha Diagnostic International Inc.). Immunoreactivity was detected using the following antibodies in the Histofine Simple StainMAX-PO secondary antibody system (Nichirei) according to the manufacturers’ instructions, donkey anti-goat Alexa Fluor 488 (1:100; Invitrogen), and donkey anti-rabbit Alexa Fluor 594 (1:100; Invitrogen), incubated for 1 h at room temperature.

### Immunohistochemical analysis

For each OE, three coronal sections located at 500 μm intervals between the caudal and rostral OE were examined. The OE contains three cell types: OSNs, supporting cells, and basal stem cells. We defined supporting cells as columnar cells located more apically in the OE and basal cells as rectangular cells lying on the lamina propria. The remaining cells were defined as OSNs. The number of OSNs labeled by anti-OMP, anti-NQO1, anti-caspase-3, and anti-8-OHdG antibodies and the number of basal cells labeled by anti-Ki67 were quantified in sections with single or double immunostaining for each antigen and counterstaining with DAPI or hematoxylin. We considered cells immunopositive if they showed staining intensity exceeding two standard deviations (SD) of the mean background intensity of the connective tissue under the lamina propria.

Coronal sections of the OE were divided into medial and lateral areas between the most lateral region of the OE and the nasal septum. The lateral and medial areas were further divided into upper and lower regions between the most dorsal and ventral edges of the OE. This resulted in four areas in each coronal OE section: dorsolateral (DL), dorsomedial (DM), ventrolateral (VL), and ventromedial (VM). The upper nasal septum region and lower nasal septum region of the DM and VM areas, respectively, were selected as areas for analysis. The number of OSNs and immunopositive (OMP-, NQO1-, Ki67-, caspase-3-, and 8-OHdG-positive) cells in 300 μm of each area or zone were counted on both right and left sides. The mean ± SD number of OSNs and OMP-, Ki67-, caspase-3-, and 8-OHdG-positive cells were counted for each group per 100 μm OE length.

To analyze the border between NQO1-positive and -negative areas (Fig. 4), NQO1-negative (negative S) and NQO1-positive (positive S) squares were identified. A NQO1-negative square was defined as a 30 mm square closest to the border with staining intensity lower than 2 SD below the mean NQO1 intensity measured in the NQO1-positive area of the OE. A NQO1-positive square was defined as a 30 mm square adjacent to the NQO1-negative square. Then, the signal intensity of OMP staining within the NQO1-positive and -negative squares was calculated. To calculate the OMP staining intensity, three coronal sections were selected in each OB and ≥5 glomeruli were randomly selected from NQO1-positive and -negative areas (Fig. 5b,c). We defined a significantly OMP-stained area as one with staining intensity >2 SD of the mean background intensity in the external plexiform layer of the OB. We calculated the percentage of significantly OMP-stained areas within a glomerulus by dividing the area of OMP staining by the total glomerulus area [(OMP-stained area/glomerulus area) × 100)]. All immunostaining analyses were performed in ImageJ (NIH).

To analyze anti-8-OHdG and anti-NQO1 or anti-8-OHdG and anti-caspase-3 co-staining (Fig. 8c,d), we counted the number of double-positive cells in the dorsal zone for each group using a fluorescence microscope at a magnification of 40×. A cell was considered a double immunopositive cell when its staining intensity, exceeding two standard deviations (SD) of the mean background intensity of the connective tissue under the lamina propria, with anti-NQO1, anti-caspase-3, or anti-8-OHdG antibodies was identical to that obtained with two different antibodies, and DAPI stained. Cells with unclear nucleus staining with DAPI were excluded from the analysis. In analysis of co-staining, we counted cells within the whole dorsal zone. To quantify MnSOD activity in the OE, the intensity of anti-MnSOD immunostaining was measured. The mean signal intensity of seven OSNs was calculated in the dorsal zone in control, Ex and CR mice (dotted circles, Fig. 8e), using seven axon bundles as the control area. The relative signal intensity of MnSOD-stained areas was calculated within the dorsal zone by dividing the mean signal intensity in the OSNs by the mean signal intensity in the axon bundles [(signal intensity of MnSOD in OSNs/signal intensity of MnSOD in axon bundles) × 100]. Signal intensity analysis was performed in ImageJ (NIH).

### Odor-induced c-fos expression in the OB

Odor-induced c-fos expression was determined as described previously^21^. Briefly, mice were placed individually in isolation boxes and supplied with pure air that was deodorized through a charcoal filter. The mice were kept in new cages without food pellets for 4 h before odor application (n = 3 mice per group). Odorants included aldehydes, lactones, and esters, and were diluted 1/10 with mineral oil. A cotton sheet soaked with 100 μL of the diluted solution was placed in a dish in the cage for 2× 1 h with a 10 min interval between placements. Following the second odor application, mice were perfused with fixative and the expression of c-fos was examined in the OB. Both primary antibodies were derived from the same animal species; therefore, two adjacent slices (4 μm interval) were selected for immunostaining with anti-NQO1 and anti-c-fos antibodies, respectively. The dorsal domain (NQO1-positive) and the ventral domain (NQO1-negative) were identified in the OB and c-fos-positive cells were counted with reference to the domain structure. C-fos-positive cells were counted in each domain under a fluorescence microscope at a magnification of 40×, with results expressed as the number of c-fos-positive cells·mm^−2^.

### Statistical analysis

The Mann-Whitney U test was used to test for differences between control and Ex and CR mice and between control and Pre mice (Figs 1e, 2b,c,e,h, 3d,e,h,i, 4d,g, 5c,d,f, 6e,f, 7b,d, 8b,f). The steel test was used to examine for differences between 2 month (Pre) and 5 month mice, and between 2 month (Pre) and 10 month mice (Fig. 1d). Error bars indicate mean ± SD. P < 0.05 was considered statistically significant.

### Study approval

All animal studies were approved by the Experimental Animal Research Committee at the University of Tokyo, and the animal study methods were carried out in accordance with the approved guidelines.
